# Correlates of gastroenterology health-services utilization among patients with gastroesophageal reflux disease: a large database analysis

**DOI:** 10.1186/s13584-019-0335-3

**Published:** 2019-08-20

**Authors:** Wasef Na’amnih, Racheli Katz, Sophy Goren, Amir Ben-Tov, Tomer Ziv-Baran, Gabriel Chodick, Khitam Muhsen

**Affiliations:** 1 Department of Epidemiology and Preventive Medicine, School of Public Health, Sackler Faculty of Medicine, Tel Aviv University, 6997801 Tel Aviv, Israel; 2 Maccabi Healthcare Services, Tel Aviv, Israel

**Keywords:** Gastroscopy, Gastroenterology health services, Gastroesophageal reflux disease, Primary care, Physicians’ board certification

## Abstract

**Background:**

Gastroesophageal reflux disease (GERD) is associated with high utilization of health care services. Diagnostic tests usually are not required to establish GERD diagnosis, but endoscopy is recommended for patients with alarm symptoms such as dysphagia and unintentional weight loss, and those whose symptoms are not relieved by proton pump inhibitors (PPIs) therapy. Evidence on the correlates of utilization of gastroenterology health services among GERD patients is limited. The study aim was to examine associations of patient and physician’s characteristics with high utilization of gastroenterology services.

**Methods:**

In a cross-sectional study using the database of the second largest integrated care organization in Israel, data of all adult GERD patients (*N* = 75,219) in 2012–2015 were analyzed. High utilization of services was assessed using two dependent variables analyzed separately: undergoing two or more gastroscopies or having six or more visits to a gastroenterology consultant during the study-period.

**Results:**

Overall, 11,261 (15.0%) patients had two or more gastroscopies and 23,703 (31.5%) had six or more visits to a gastroenterology consultant. The likelihood of high utilization of gastroscopy increased with age; in immigrants from the Former Soviet Union versus patients who were born in Israel; residents of Jerusalem, the south, the north and Haifa districts versus the center district; in patients with high PPI purchases, and in patients who belonged to clinics in which the physician-manger had no board certification. The correlates were similar for visits to a gastroenterology consultant.

**Conclusions:**

Patient and physician’s characteristics were related to high utilization of gastroenterology services among GERD patients. The associations with age and country of birth might reflect more severe disease. The regional differences warrant further research and interventions at the district level. Training in gastroenterology of primary care physicians without a board certification is warranted.

**Electronic supplementary material:**

The online version of this article (10.1186/s13584-019-0335-3) contains supplementary material, which is available to authorized users.

## Background

Gastroesophageal reflux disease (GERD) is common worldwide [1]. Complications of GERD include erosive esophagitis (EE), Barrett’s esophagus (BE) [2] and esophageal adenocarcinoma [3]. Diagnostic tests usually are not required to establish GERD diagnosis [3], but endoscopy is recommended for patients with alarm symptoms such as dysphagia, unintentional weight loss and anemia, and those whose symptoms are not relieved by proton pump inhibitors (PPIs) therapy [3, 4].

GERD negatively affects quality of life and causes substantial economic burden to the health care system and reduced work productivity, especially in patients with severe and frequent symptoms [5–8]. The economic burden attributed to GERD is driven by consultations costs, diagnostic tests and medications [9–11]. Most studies on utilization of health care services among GERD patients assessed general services such as hospitalizations, emergency department visits and physician visits [5, 6, 8, 12], mostly according to intensity and severity of symptoms [5–8], with limited or no adjustment for patient’s characteristics (such as demographics and comorbidity) or physician’s characteristics [5, 6, 8]. Only few studies assessed utilization of gastroenterology health services among GERD patients [10, 13]. A study on repeated upper endoscopy in the Veterans Health Administration included 14,284 patients with reflux [13], indicated that 54.2 and 31.5% of the repeated endoscopy in this group were classified as probable and possible overuse, respectively, while only 14.3% likely represented appropriate use. Understanding the correlates of high utilization of gastroenterology health services among GERD patients may be the first step towards efficient, cost-saving and safe treatment. Accordingly, the aim of the current study was to examine associations of patient and physician’s characteristics with high utilization of gastroenterology services (outpatient visits to gastroenterology consultant and gastroscopy) among patients with GERD.

## Methods

### Study design and population

A cross-sectional study was conducted using the computerized databases of Maccabi Healthcare Services (MHS), the second largest integrated care organization in Israel. MHS currently has over 2 million members, comprising about 25% of Israel’s population. Data of all patients aged more than 18 years with GERD between January 1, 2012 and December 31, 2015, were analyzed. Patients with GERD were identified using physician’s diagnosis code of the International Classification of Diseases, 9th edition (ICD-9) for GERD (530.81) or MHS corresponding codes (Y14968 code for esophageal reflux, gastroesophageal reflux disease, GERD, and reflux esophageal).

### The dependent variables

High utilization of gastroenterology services was defined as: 1) undergoing two or more gastroscopies; and 2) six or more (upper tertile) visits to gastroenterology consultant, during the four-year study period. The diagnosis of uncomplicated GERD usually does not require gastroscopy [4]. In the study sample, 42.6% of GERD patients underwent at least one diagnostic gastroscopy during the study period: 27.7% underwent one gastroscopy and 15.0% underwent two or more gastroscopies. Accordingly, we considered that undergoing two or more gastroscopies during the study period as high utilization of gastroscopy.

### The independent variables

The selection of the independent variables was based on our hypothesis that characteristics of both patients (e.g., age, comorbidity), and physicians (e.g., board certification), are related to utilization of these services. This was stimulated by previous studies on associations of demographic and clinical factors with health care utilization [6, 12, 14, 15].

### Patient’s characteristics

Data were obtained on age (in years, categorized as 19–34, 35–44, 45–54, 55–64, 65–74, 75+), sex, residential district and country of birth (grouped as Israel, Former Soviet Union [FSU], Europe/Americas, Asia/North Africa and other/unknown). Socioeconomic status (SES) of the town of residence defined by the Central Bureau of Statistic [16] was used as a proxy of SES. Patients who lived in towns with SES ranks of 1–4 and 5–10 were classified as living in low and middle/high SES communities, respectively. MHS registries were used to determine the presence of diabetes mellitus [17], hypertension [18] and cardiovascular disease [19]. Information was obtained on purchasing PPIs; patients were classified as high users if they had above the median number (eight) of PPIs purchases in the study sample.

### Characteristics of primary care physicians

A primary care clinic can include several physicians who treat patients, and a manger (mostly one of the primary care physicians in the clinic). We extracted information on both the treating primary care physician and the physician-manager of the clinic. Data were obtained regarding the physician’s board certification (none, family medicine, internal medicine, medical director and other) and physician’s seniority (categorized as having above the median number of seniority years versus having the median or less of seniority years).

### Statistical analysis

Differences between patients with high utilization of gastroscopy (undergoing two or more tests) and those who one or no gastroscopy, in demographic and clinical characteristics were assessed using the chi-square test. Multivariable analysis was performed using logistic regression models. Similar analyses were performed for the dependent variable high utilization of visits to a gastroenterology consultant (six or more). Unadjusted and adjusted odds ratios (and 95% confidence intervals) for each independent variable were obtained from logistic regression models. Statistical significance was set at *P* < 0.05. Data were analyzed using SPSS version 25 (IBM, New York, United States).

## Results

We identified 75,219 patients (57.1% females) with GERD with a mean age of 53.1 years (standard deviation 15.9). Additional demographic characteristics are presented in Additional file 1: Table S1. Overall, 11,261 (15.0%) underwent two or more gastroscopies and 23,703 (31.5%) had six or more visits to a specialist in gastroenterology.

### Factors associated with high utilization of gastroscopy (two or more tests)

The percentage of patients aged 55 years or older was higher in those who had two gastroscopies or more than in patients who had 0–1 gastroscopies. The group of high utilization of gastroscopy included also higher percentages of patients who were born in the FSU, residents of Jerusalem and the south districts; and patients with heart disease, diabetes mellitus, hypertension and high PPIs purchases compared to the group who performed 0–1 gastroscopies. The percentage of physician-managers who did not have a board certification was higher in the high utilization group (Table 1).

**Table 1:** Utilization of gastroscopy (two tests or more) according to patient and physician’s characteristics

Variable	≥2 gastroscopies *N* = 11,261	0–1 gastroscopies *N* = 63,958	*P* value
Sex, female	6570 (58.3)	36,363 (56.9)	0.003
Age, years		Df = 5	trend < 0.001
19–34	713 (6.3)	10,081 (15.8)	< 0.001
35–44	1250 (11.1)	11,541 (18.0)	
45–54	2384 (21.2)	12,850 (20.1)	
55–64	3231 (28.7)	13,744 (21.5)	
65–74	2534 (22.5)	10,038 (15.7)	
75+	1149 (10.2)	5704 (8.9)	
Country of birth		Df = 4	< 0.001
Israel	5701 (50.6)	40,163 (62.8)	
Former Soviet Union	3782 (33.6)	13,893 (21.7)	
Asia/North Africa	614 (5.5)	3325 (5.2)	
Europe/Americas	806 (7.2)	4701 (7.4)	
Other/ unknown	358 (3.2)	1876 (2.9)	
Residential district		Df = 5	< 0.001
Center	3000 (26.7)	20,866 (32.7)	
Jerusalem	735 (6.5)	2561 (4.0)	
North	1051 (9.4)	4827 (7.6)	
Haifa	1209 (10.8)	5582 (8.8)	
Tel Aviv	2152 (19.2)	20,344 (31.9)	
South	2798 (24.9)	8183 (12.8)	
SES of place of residence			< 0.001
Middle/High (5–10)	8671 (80.8)	51,196 (84.3)	
Low (1–4)	2067 (19.2)	9552 (15.7)	
Background diseases			
Heart disease	2391 (21.2)	9665 (15.1)	< 0.001
Hypertension	5313 (47.2)	21,935 (34.3)	< 0.001
Diabetes	2151 (19.1)	8144 (12.7)	< 0.001
Number of PPIs purchases			< 0.001
Low (0–8)	3459 (30.7)	35,575 (55.6)	
High (9+)	7802 (69.3)	28,383 (44.4)	
Primary care physician board certification		Df = 3	< 0.001
Family medicine	2961 (26.3)	18,377 (28.7)	
None	4562 (40.5)	23,652 (37.0)	
Internal medicine	2702 (24.0)	15,382 (24.1)	
Other	1034 (9.2)	6543 (10.2)	
Board certification of the clinic physician-manager		Df = 4	< 0.001
Family medicine	1700 (15.1)	10,130 (15.9)	
None	1400 (12.5)	4725 (7.4)	
Internal medicine	1189 (10.6)	7452 (11.7)	
Medical director	4440 (39.6)	26,189 (41.1)	
Other	2493 (22.2)	15,250 (23.9)	
Primary care physician seniority			0.001
Low (0–8 years)	5830 (51.8)	31,984 (50.0)	
High (9–43 years)	5429 (48.2)	31,970 (50.0)	

Data presented are numbers and percentages in parenthesis

*Df* degrees of freedom, *PPIs* proton pump inhibitors, *SES* socioeconomic status

Missing data: Residential district (205 patients); SES (3733 patients)

A multivariable analysis showed that compared to patients aged 19–34 years, the likelihood of high utilization of gastroscopy significantly increased with age. Patients who were born in FSU had 1.39-fold increased likelihood for high utilization of gastroscopy than those who were born in Israel. Compared to residents of the center district, residents of Jerusalem and the south districts had more than 2-fold higher likelihood for gastroscopy high utilization and patients who lived in Haifa and north districts had ~ 1.50-fold increased likelihood. Patients with high number of PPIs purchases had 2.43-fold higher likelihood for gastroscopy high utilization compared to patients who had less PPIs purchases. Having a clinic physician-manager without a board certification was associated with 1.27-fold higher likelihood of gastroscopy utilization compared to having a physician-manager with board certification in family medicine (Table 2).

**Table 2:** Unadjusted and adjusted associations of patient and physician’s characteristics with high utilization of gastroscopy

Variable	Unadjusted OR (95% CI)	*P* value	Adjusted OR (95% CI)	*P* value*
Sex, females vs. males	1.06 (1.02–1.11)	0.003	0.99 (0.95–1.04)	0.7
Age, years	Df = 5	< 0.001	Df = 5	< 0.001
19–34	Reference		Reference	
35–44	1.53 (1.39–1.69)	< 0.001	1.33 (1.20–1.47)	< 0.001
45–54	2.62 (2.40–2.86)	< 0.001	2.03 (1.85–2.23)	< 0.001
55–64	3.32 (3.05–3.62)	< 0.001	2.30 (2.09–2.52)	< 0.001
65–74	3.57 (3.27–3.90)	< 0.001	2.22 (2.01–2.46)	< 0.001
75+	2.85 (2.58–3.14)	< 0.001	1.55 (1.38–1.74)	< 0.001
Country of birth	Df = 4	< 0.001	Df = 4	< 0.001
Israel	Reference		Reference	
Former Soviet Union	1.92 (1.83–2.01)	< 0.001	1.39 (1.32–1.46)	< 0.001
Asia/North Africa	1.30 (1.19–1.42)	< 0.001	1.01 (0.92–1.12)	0.8
Europe/Americas	1.21 (1.12–1.31)	< 0.001	0.92 (0.84–1.00)	0.05
Other/ unknown	1.34 (1.20–1.51)	< 0.001	1.02 (0.90–1.16)	0.7
Residential district	Df = 5	< 0.001	Df = 5	< 0.001
Center	Reference		Reference	
Jerusalem	2.00 (1.82–2.19)	< 0.001	2.27 (2.02–2.54)	< 0.001
North	1.51 (1.40–1.64)	< 0.001	1.49 (1.36–1.63)	< 0.001
Haifa	1.51 (1.40–1.62)	< 0.001	1.44 (1.32–1.57)	< 0.001
Tel Aviv	0.74 (0.69–0.80)	< 0.001	0.73 (0.68–0.78)	< 0.001
South	2.38 (2.25–2.52)	< 0.001	2.43 (2.26–2.61)	< 0.001
SES of place of residence				
Middle/High (5–10)	Reference		Reference	
Low (1–4)	1.28 (1.21–1.35)	< 0.001	1.04 (0.97–1.12)	0.25
Background diseases				
Heart disease (reference = no)	1.51 (1.44–1.59)	< 0.001	1.12 (1.05–1.18)	< 0.001
Hypertension (reference = no)	1.71 (1.64–1.78)	< 0.001	1.01 (0.96–1.06)	0.7
Diabetes (reference = no)	1.62 (1.54–1.71)	< 0.001	1.14 (1.07–1.21)	< 0.001
Number of PPIs purchases				
Low (0–8)	Reference		Reference	
High (9+)	2.83 (2.71–2.95)	< 0.001	2.43 (2.31–2.54)	< 0.001
Primary care physician board certification	Df = 3	< 0.001	Df = 3	< 0.001
Family medicine	Reference		Reference	
None	1.20 (1.14–1.26)	< 0.001	0.94 (0.88–1.00)	0.03
Internal medicine	1.09 (1.03–1.15)	0.003	1.02 (0.96–1.08)	0.6
Other	1.22 (1.06–1.40)	0.005	1.14 (0.98–1.32)	0.09
Board certification of the clinic physician-manager	Df = 4	< 0.001	Df = 4	< 0.001
Family medicine	Reference		Reference	
None	1.77 (1.63–1.91)	< 0.001	1.27 (1.16–1.39)	< 0.001
Internal medicine	0.95 (0.88–1.03)	0.2	0.95 (0.87–1.03)	0.2
Medical director	1.01 (0.95–1.07)	0.7	0.90 (0.83–0.97)	0.004
Other	0.91 (0.85–0.98)	0.009	1.05 (0.96–1.14)	0.3
Primary care physician seniority				
Low (0–8 years)	Reference		Reference	
High (9–43 years)	0.93 (0.90–0.97)	0.001	0.99 (0.94–1.04)	0.7

*CI* confidence interval, *Df* degrees of freedom, *OR* odds ratio, *PPIs* proton pump inhibitors, *SES* socioeconomic status

Missing data: Residential district (205 patients); SES (3733 patients)

### Factors associated with having six or more visits to a gastroenterology consultant

A higher percentage of females and older patients was found among those with high number (six or more) of visits to a gastroenterology consultant than in patients who had less visits. The former group included also higher percentages of patients who were born in Asia/North Africa and who had a high number of PPIs purchases (Table 3).

**Table 3:** Utilization of visits to gastroenterology a consultant according to patients and physicians’ characteristics

Variable	High number of visits (≥6) N (%)	Low number of visits (0–5) N (%)	*P* value
Total	23,703	51,510	
Sex, female	14,621 (61.7)	28,312 (55.0)	< 0.001
Age, years		Df = 5	< 0.001
19–34	2466 (10.4)	8328 (16.2)	
35–44	3010 (12.7)	9781 (19.0)	
45–54	3839 (16.2)	11,395 (22.1)	
55–64	5477 (23.1)	11,498 (22.3)	
65–74	5250 (22.1)	7322 (14.2)	
75+	3662 (15.4)	3191 (6.2)	
Country of Birth		Df = 4	< 0.001
Israel	13,664 (57.6)	32,200 (62.5)	
Former Soviet Union	5404 (22.8)	12,271 (23.8)	
Asia/North Africa	1700 (7.2)	2239 (4.3)	
Europe/America	2093 (8.8)	3414 (6.6)	
Other/ unknown	843 (3.6)	1391 (2.7)	
Residential district		Df = 5	< 0.001
Center	7099 (30.0)	16,767 (32.6)	
Jerusalem	1602 (6.8)	1694 (3.3)	
North	1683 (7.1)	4195 (8.2)	
Haifa	1782 (7.5)	5009 (9.7)	
Tel Aviv	6942 (29.4)	15,554 (30.3)	
South	3908 (16.5)	7073 (13.8)	
SES of place of residence			< 0.001
Middle/High (5–10)	18,324 (81.0)	41,543 (85.0)	
Low (1–4)	4285 (19.0)	7334 (15.0)	
Background diseases			
Heart disease	6483 (27.3)	5573 (10.8)	< 0.001
Hypertension	11,226 (47.4)	16,022 (31.1)	< 0.001
Diabetes	4669 (19.7)	5626 (10.9)	< 0.001
Number of PPIs purchases			< 0.001
Low (0–8)	9641 (40.7)	29,393 (57.1)	
High (9+)	14,063 (59.3)	22,122 (42.9)	
Primary care physician board certification		Df = 3	< 0.001
Family medicine	6673 (28.2)	14,665 (28.5)	
None	8896 (37.5)	19,318 (37.5)	
Internal medicine	5599 (23.6)	12,485 (24.2)	
Other	2535 (10.7)	5042 (9.8)	
Board certification of the clinic physician-manager		Df = 4	< 0.001
Family medicine	3800 (16.1)	8030 (15.6)	
None	2361 (10.0)	3764 (7.3)	
Internal medicine	2589 (11.0)	6052 (11.8)	
Medical director	9446 (40.0)	21,183 (41.2)	
Other	5409 (22.9)	12,334 (24.0)	
Primary care physician seniority			0.001
Low (0–8 years)	12,125 (51.2)	25,689 (49.9)	
High (9–43 years)	11,578 (48.8)	25,821 (50.1)	

*Df* degrees of freedom, *PPIs* proton pump inhibitors, *SES* socioeconomic status

Missing data: Residential district (205 patients); SES (3733 patients)

The strength of these associations was mostly attenuated in multivariable model (Table 4).

**Table 4:** Associations of patient and physician’s characteristics with high utilization of visits to a gastroenterology consultant

Variable	Unadjusted OR (95% CI)	*P* value	Adjusted OR (95% CI)	*P* value*
Sex, females vs. males	1.32 (1.28–1.36)	< 0.001	1.43 (1.38–1.48)	< 0.001
Age, years	Df = 5	< 0.001	Df = 5	< 0.001
19–34	Reference		Reference	
35–44	1.04 (0.98–1.10)	0.2	1.00 (0.94–1.06)	0.9
45–54	1.14 (1.07–1.21)	< 0.001	0.96 (0.90–1.02)	0.2
55–64	1.61 (1.52–1.70)	< 0.001	1.12 (1.05–1.19)	0.001
65–74	2.42 (2.29–2.56)	< 0.001	1.39 (1.30–1.49)	< 0.001
75+	3.88 (3.63–4.14)	< 0.001	1.81 (1.66–1.96)	< 0.001
Country of birth	Df = 4	< 0.001	Df = 4	< 0.001
Israel	Reference		Reference	
Former Soviet Union	1.04 (0.99–1.08)	0.054	0.77 (0.74–0.81)	< 0.001
Asia/North Africa	1.79 (1.68–1.91)	< 0.001	1.21 (1.13–1.30)	< 0.001
Europe/Americas	1.45 (1.36–1.53)	< 0.001	0.92 (0.86–0.98)	0.01
Other/ unknown	1.43 (1.31–1.56)	< 0.001	0.99 (0.90–1.09)	0.9
Residential district	Df = 5	< 0.001	Df = 5	< 0.001
Center	Reference		Reference	
Jerusalem	2.23 (2.07–2.41)	< 0.001	2.10 (1.92–2.30)	< 0.001
North	0.95 (0.89–1.01)	0.09	0.81 (0.75–0.88)	< 0.001
Haifa	0.84 (0.79–0.89)	< 0.001	0.80 (0.74–0.86)	< 0.001
Tel Aviv	1.05 (1.01–1.09)	0.009	0.99 (0.94–1.05)	0.8
South	1.31 (1.24–1.37)	< 0.001	1.24 (1.17–1.31)	< 0.001
SES of place of residence				
Middle/High (5–10)	Reference		Reference	
Low (1–4)	1.33 (1.27–1.38)	< 0.001	0.85 (0.80–0.89)	< 0.001
Background diseases				
Heart disease (reference = no)	3.10 (2.98–3.23)	< 0.001	2.28 (2.18–2.39)	< 0.001
Hypertension (reference = no)	1.99 (1.93–2.06)	< 0.001	1.24 (1.19–1.29)	< 0.001
Diabetes (reference = no)	2.00 (1.92–2.09)	< 0.001	1.28 (1.22–1.35)	< 0.001
Number of PPIs purchases				
Low (0–8)	Reference		Reference	
High (9+)	1.94 (1.88–2.00)	< 0.001	1.47 (1.42–1.53)	< 0.001
Primary care physician board certification	Df = 3	< 0.001	Df = 3	< 0.001
Family medicine	Reference		Reference	
None	0.99 (0.95–1.03)	0.5	0.95 (0.90–0.99)	0.01
Internal medicine	0.97 (0.93–1.01)	0.2	0.99 (0.94–1.04)	0.7
Other	0.97 (0.87–1.09)	0.6	0.99 (0.88–1.12)	0.9
Board certification of the clinic physician-manager	Df = 4	< 0.001	Df = 4	< 0.001
Family medicine	Reference		Reference	
None	1.33 (1.24–1.41)	< 0.001	1.47 (1.37–1.59)	< 0.001
Internal medicine	0.90 (0.85–0.96)	0.001	0.94 (0.88–1.00)	0.05
Medical Director	0.94 (0.90–0.99)	0.01	0.99 (0.94–1.05)	0.8
Other	0.90 (0.86–0.95)	< 0.001	0.96 (0.90–1.02)	0.2
Primary care physician seniority				
Low (0–8 years)	Reference		Reference	
High (9–43 years)	0.95 (0.92–0.98)	0.001	0.98 (0.94–1.02)	0.3

*CI* confidence interval, *Df* degrees of freedom, *OR* odds ratio, *PPIs* proton pump inhibitors, *SES* socioeconomic status

Missing data: Residential district (205 patients); SES (3733 patients)

## Discussion

We found that the utilization of gastroscopy and/or visits to a gastroenterology consultant by GERD patients increased with age. There was a higher utilization by patients in the peripheral districts than in the center of Israel; by patients born in the FSU than in those born in Israel; by patients with heart disease, diabetes and hypertension; and by patients of primary care clinics headed by non-board certified physicians.

The finding that utilization increased with patients’ age is probably explained by physicians’ concerns of GERD complications such as EE, BE, and esophageal cancer that increase with age [20–23], However, this is an unlikely explanation for the observed higher utilization of gastroenterology services by patients from the periphery compared with those from the center of Israel. Regional differences in the severity and complications of GERD are not expected. Therefore, these differences probably reflect variation in referral policy across districts and warrant further exploration. A study from the United States on repeated upper endoscopy in general, showed similar regional differences, even after controlling for diagnostic codes of gastroesophageal illnesses [14].

Patients born in the FSU and in Asia and North Africa utilized gastroscopy more than patients who were born in Israel. This might be due to differences in the severity of GERD and/or its complications. Indeed, it has been shown that Israelis born in FSU and Israelis who emigrated from Asian countries (mostly west Asia) display higher risk for gastroesophageal adenocarcinoma than persons born in Israel [24]. Ethnic differences in esophageal pathology in patients undergoing endoscopy were also reported in the United States [25–28].

The association between the number of PPIs purchases and utilization of services is probably due to the intensity of GERD symptoms [8]. It is consistent with the observation by Mody et al. [29] that twice-daily PPIs use was associated with higher health services utilization and costs than once-daily use. The association of having heart disease, diabetes, hypertension and high number with visits to a gastroenterology consultant might be attributed to medical surveillance. The association with heart disease might be related to GERD symptoms involving chest pain in some patients. PPIs failure is common among diabetic patients [15] and this might explain the positive association of diabetes and utilization of services.

While health care utilization patterns and resulting costs are affected by the severity of GERD symptoms [5] and comorbidity, we also found that physician’s education and training have a role in the management of the disease. In the Israeli system, referrals such as gastroscopy require approval of the physician-manager. Therefore, additional education or training of physician-managers who do not have a formal board certification in areas of family medicine/gastroenterology might be warranted to improve care and reduce cost related to GERD management.

The main strength of our study is its use of multi-year data of a large sample of adult GERD patients, who were identified by their diagnostic code. The code of GERD was partially validated by the purchases of PPIs by most patients at least once during the study period. However, the use of data from medical records of MHS database over a four-year study period has limitations. Differences might exist between physicians in documenting medical information. Information on the indications of gastroscopy and the results of the tests are lacking and we cannot determine whether the referrals were clinically appropriate or represented overuse of services. Therefore, our findings refer to correlates with high utilization of gastroenterology services rather than overuse.

## Conclusions

Both patient and physician’s characteristics play a role in high utilization of gastroenterology health services among GERD patients. The relationships with age and country of birth might reflect more severe disease in older people and some ethnic groups. The regional differences warrant further research and interventions at the district level. Training in gastroenterology of primary care physicians without a board certification is warranted.

## Additional file

Additional file 1: **Table S1.** Characteristics of patients with GERD (*N* = 75,219), 2012–2015. (DOCX 15 kb)

## Data Availability

Individual level data from this study cannot be made publically available due to legal and ethical restrictions.

## Abbreviations

BE: Barrett’s esophagus
FSU: Former Soviet Union
GERD: Gastroesophageal reflux disease
ICD-9: The International Classification of Diseases, 9th edition
MHS: Maccabi healthcare services
PPIs: Proton pump inhibitors
SES: Socioeconomic status
