# Supplementary material for: Correlates of gastroenterology health-services utilization among patients with gastroesophageal reflux disease: a large database analysis
Source: Isr J Health Policy Res. 2019 Aug 20;8:66. doi: 10.1186/s13584-019-0335-3 (PMC6700833; doi:10.1186/s13584-019-0335-3)
Supplement: Supplementary file 1 — Table S1. Characteristics of patients with GERD (N = 75,219), 2012–2015. (DOCX 15 kb) [file 13584_2019_335_MOESM1_ESM.docx]

**Table S1.** Characteristics of patients with GERD (N=75,219), 2012-2015

| **Variable** | **Number of patients, (%)** |
| --- | --- |
| **Sex, female** | 42,933 (57.1) |
| **Age, years** |  |
| 19-34 | 10,794 (14.4) |
| 35-44 | 12,791 (17.0) |
| 45-54 | 15,234 (20.3) |
| 55-64 | 16,975 (22.6) |
| 65-74 | 12,572 (16.7) |
| 75+ | 6,853 (9.1) |
| **Country of birth** |  |
| Israel | 45,864 (61.0) |
| Former Soviet Union | 17,675 (23.5) |
| Asia/North Africa | 3,939 (5.2) |
| Europe/Americas | 5,507 (7.3) |
| Other/ unknown | 2,234 (3.0) |
| **Residential district** |  |
| Jerusalem | 3,296 (4.4) |
| North | 5,878 (7.8) |
| Haifa | 6,791 (9.0) |
| Center | 23,866 (31.7) |
| Tel Aviv | 22,496 (29.9) |
| South | 10,981 (14.6) |
| **SES of place of residence** |  |
| Middle/High (5-10) | 59,867 (79.6) |
| Low (1-4) | 11,619 (15.4) |

GERD: Gastro-esophageal reflux disease; PPIs: proton pump inhibitors; SES: socioeconomic status.
